# Escherichia coli Pyruvate Dehydrogenase Complex Is an Important Component of CXCL10-Mediated Antimicrobial Activity

**DOI:** 10.1128/IAI.00552-15

**Published:** 2015-12-28

**Authors:** Kirsten M. Schutte, Debra J. Fisher, Marie D. Burdick, Borna Mehrad, Amy J. Mathers, Barbara J. Mann, Robert K. Nakamoto, Molly A. Hughes

**Affiliations:** a Department of Medicine, Division of Infectious Diseases and International Health, University of Virginia, Charlottesville, Virginia, USA; b Department of Medicine, Division of Pulmonary and Critical Care Medicine, University of Virginia, Charlottesville, Virginia, USA; c Department of Molecular Physiology and Biological Physics, University of Virginia, Charlottesville, Virginia, USA

## Abstract

Chemokines are best recognized for their role within the innate immune system as chemotactic cytokines, signaling and recruiting host immune cells to sites of infection. Certain chemokines, such as CXCL10, have been found to play an additional role in innate immunity, mediating CXCR3-independent killing of a diverse array of pathogenic microorganisms. While this is still not clearly understood, elucidating the mechanisms underlying chemokine-mediated antimicrobial activity may facilitate the development of novel therapeutic strategies effective against antibiotic-resistant Gram-negative pathogens. Here, we show that CXCL10 exerts antibacterial effects on clinical and laboratory strains of Escherichia coli and report that disruption of pyruvate dehydrogenase complex (PDHc), which converts pyruvate to acetyl coenzyme A, enables E. coli to resist these antimicrobial effects. Through generation and screening of a transposon mutant library, we identified two mutants with increased resistance to CXCL10, both with unique disruptions of the gene encoding the E1 subunit of PDHc, *aceE*. Resistance to CXCL10 also occurred following deletion of either *aceF* or *lpdA*, genes that encode the remaining two subunits of PDHc. Although PDHc resides within the bacterial cytosol, electron microscopy revealed localization of immunogold-labeled CXCL10 to the bacterial cell surface in both the E. coli parent and *aceE* deletion mutant strains. Taken together, our findings suggest that while CXCL10 interacts with an as-yet-unidentified component on the cell surface, PDHc is an important mediator of killing by CXCL10. To our knowledge, this is the first description of PDHc as a key bacterial component involved in the antibacterial effect of a chemokine.

## INTRODUCTION

Chemokines are small (8- to 12-kDa) proteins originally recognized for their ability to act as cellular messengers that promote cell differentiation, activation, and migration, such as routing leukocytes to areas of inflammation as part of the innate immune response (1). More recently, they have been recognized for their diverse roles in many different biological processes, not only participating in the innate and adaptive immune response but also acting in such disparate processes as angiogenesis, hematopoiesis, organogenesis, autoimmunity, and tumor metastasis (2, 3). Such effects are mainly achieved through the binding of chemokines to receptors on cellular surfaces and the subsequent initiation of intracellular signaling cascades (4). In addition to processes activated through ligand-receptor interactions, a growing number of chemokines have been observed to have host receptor-independent antimicrobial effects against a diverse range of bacteria and fungi (5, 6). The interferon-inducible, glutamic acid-leucine-arginine-negative (ELR^−^) CXC chemokines, CXCL9, CXCL10, and CXCL11, in particular have been found to exhibit antimicrobial properties (2, 7).

CXCL9, CXCL10, and CXCL11 are produced by resident host cells and infiltrating phagocytes within inflamed tissues in response to stimulation by proinflammatory cytokines such as interferon gamma. Signaling effects are then achieved by the interaction of these chemokines with a shared receptor, CXCR3, found mainly on T helper 1 (Th1) cells and natural killer cells (7–9). *In vitro* antimicrobial activity of the interferon-inducible ELR^−^ CXC chemokines, however, occurs in the absence of such cellular signaling (10). Direct *in vitro* killing effects have been observed against a variety of pathogenic bacteria, including Bacillus anthracis, Staphylococcus aureus, Listeria monocytogenes, Chlamydophila pneumoniae, and Escherichia coli (5, 11–13). The concentration of CXCL10 necessary to produce such effects is similar to the *in vitro* MICs of most known antimicrobial chemokines and host cationic antimicrobial proteins (CAMPs), such as defensins, which range from 0.1 to 100 μg/ml (14). *In vivo* studies support the physiologic relevance of these findings in acute infection. For example, in a murine model of inhalational anthrax using C57BL/6 mice relatively resistant to Sterne strain infection, antibody neutralization of CXCR3 or use of CXCR3-knockout mice, and hence disruption of the ligand-receptor interaction, had no deleterious effect on survival. Antibody neutralization of CXCL9 or neutralization of CXCL9 and CXCL10, however, increased associated mortality from <5% to ∼60% (10).

While the exact process by which the interferon-inducible ELR^−^ CXC cytokines mediate their antimicrobial effect remains unclear, the C terminus has been implicated in their antimicrobial activity. Interferon-inducible ELR^−^ CXC chemokines interact with CXCR3 primarily via the N-terminal region of the chemokine and amino acid residues in two loop regions (15) and, through this interaction with CXCR3, recruit additional immune cells to their location. In contrast, their positively charged C-terminal end contains an amphipathic alpha-helical moiety that is structurally similar to those of CAMPs (13). In a survey of 30 different chemokines by Yang et al. (13), the existence of not only cationic moieties but also a topographical amphipathic structure consisting of discrete groupings of hydrophilic and hydrophobic sections appeared to be a common finding among chemokines with direct antimicrobial activity—a feature that they share with defensins (13, 16). Extrapolated from the mechanism of action of many defensins, one relatively simple model of chemokine-mediated antimicrobial activity postulates that positively charged regions of antimicrobial chemokines interact with negatively charged moieties on bacterial cell surfaces, resulting in cell lysis through membrane disruption or permeabilization (13). However, recent work with B. anthracis has revealed that the bacterial protein FtsX is required for CXCL10-mediated antimicrobial activity (2). Studies investigating the mechanism of action of other antimicrobial peptides, including human defensins, support the possibility that the specific targets and mechanisms of antimicrobial action may differ among the diverse range of microorganisms affected by a given antimicrobial peptide (17, 18). The identification of such bacterial targets has the potential to open up new avenues of inquiry into the mechanisms by which chemokine-mediated antimicrobial effects occur. Similarly, such information has important implications for the development of novel therapeutic agents, including those with activity against multidrug-resistant pathogens.

We hypothesized that, similarly to what we found with B. anthracis, specific bacterial targets necessary for CXCL10-mediated killing would be present in other bacteria that were susceptible to the interferon-inducible ELR^−^ CXC chemokines. To assess the relevance of the antimicrobial effect of CXCL10 against clinically important Gram-negative bacteria such as those within the family Enterobacteriaceae, we tested a multidrug-resistant clinical strain of E. coli (a multilocus sequence type 131 [ST131] strain also known to carry *bla*_KPC_) for susceptibility to recombinant human CXCL10. Based on our previous studies (2), we used CXCL10 as a prototype chemokine to screen a transposon mutant library generated from a K-12-derived laboratory strain of E. coli to test our hypothesis and identify putative Gram-negative bacterial targets.

## MATERIALS AND METHODS

### Bacterial strains and growth conditions.

**FIG 1:**
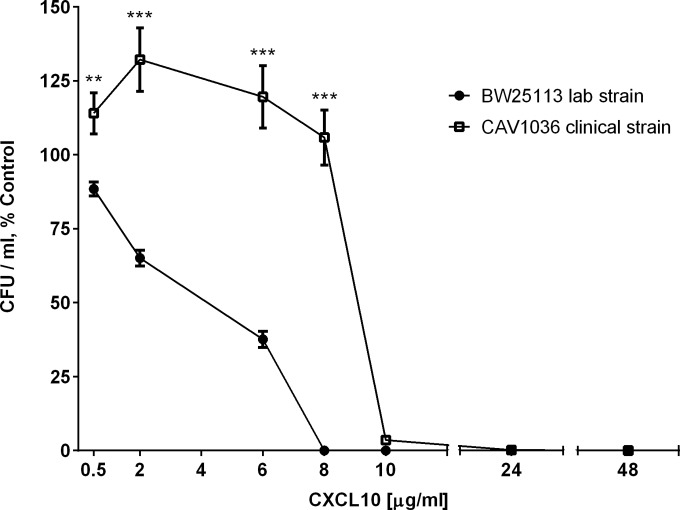
CXCL10 exhibits antimicrobial activity against both E. coli laboratory and multidrug-resistant clinical strains. The E. coli BW25113 lab strain and E. coli CAV1036, a multidrug-resistant clinical strain, were exposed to increasing concentrations of CXCL10, after which serial dilutions were plated on LB agar for CFU-per-milliliter determination following overnight incubation at 37°C. The CFU-per-milliliter data are expressed as percent control (a percentage of the viable CFU per milliliter for each specific isolate obtained by dividing the number of CFU per milliliter for the isolate after exposure to CXCL10 by the number of CFU per milliliter of that same isolate's corresponding untreated control). EC_50_ values for CXCL10 were calculated as indicated in Materials and Methods. The EC_50_ against the E. coli BW25113 lab strain was 2.8 μg/ml CXCL10 (95% CI, 2.6 to 3.1). The EC_50_ against the CAV1036 clinical isolate was 8.6 μg/ml CXCL10 (95% CI, 7.9 to 9.3). Data points represent mean values ± standard errors of the means (*n* = 3 independent experiments performed in duplicate). Two-way ANOVA, *P* < 0.0001; Bonferroni multiple-comparison test, **, *P* = 0.004; ***, *P* < 0.0001.

A multidrug-resistant clinical ST131 strain of E. coli designated CAV1036, initially identified during a hospital outbreak of infections by carbapenem-resistant Enterobacteriaceae, was obtained from the University of Virginia Health System Clinical Microbiology Laboratory (19, 20). E. coli BW25113 (henceforth referred to as the parent strain throughout the text, except for being specifically designated by strain name in Fig. 1) and its Δ*aceE*, Δ*aceF*, and Δ*lpdA* derivatives (JW0110-2, JW0111-2, and JW0112-3, respectively) were obtained from the Keio Collection (21) via the Coli Genetic Stock Center (Yale University, New Haven, CT, USA). Removal of the FLP recognition target (FRT) flanking the kanamycin resistance cassette in the Δ*aceE* strain was accomplished by transforming electrocompetent cells with pCP20 (strain designation BT340; Coli Genetic Stock Center) as described previously (22). E. coli strain Alpha-select (Bioline, Taunton, MA, USA) was used for propagation of *aceE*-containing and empty cloning vector pBR322 (New England BioLabs, Ipswich, MA, USA). Bacteria were grown at 37°C with shaking (250 rpm) in Luria-Bertani (LB) broth, or “no-salt LB broth” (10 g tryptone, 5 g yeast extract per liter of distilled water [dH_2_O]) in preparation for generation of the transposon library. Overnight liquid cultures were subcultured, and log-phase bacteria were harvested at an optical density (OD) of ≈0.6 at 600 nm. The following antibiotics were added to growth medium as appropriate: ampicillin, 100 μg/ml; kanamycin, 50 μg/ml.

### Antimicrobial assays.

E. coli (≈3 × 10^5^ cells per sample well) was treated with recombinant human CXCL10 (Peprotech, Rocky Hill, NJ, USA) in sterile water stabilized with 0.3% human serum albumin (HSA; Grifols Therapeutics Inc., Research Triangle Park, NC, USA) or an equal volume of 0.3% HSA alone as vehicle. Protamine (Sigma-Aldrich, St. Louis, MO, USA) was similarly prepared. Assay conditions were as previously described (13) with modifications. Bacteria were inoculated into 10 ml LB broth and incubated overnight at 37°C in a shaking incubator. The bacterial cultures were diluted into LB broth prewarmed to 37°C to an OD of 0.1 to 0.3 at 600 nm; subcultures were then grown under the same conditions to an OD of ≈0.6 at 600 nm. These mid-log-phase bacteria were diluted to the assay concentration of ≈3 × 10^6^ cells/ml in 10 mM potassium phosphate buffer (pH 7.4) supplemented with 1% Trypticase soy broth (13). After 2 h of incubation, serial dilutions were plated on LB and incubated overnight at 37°C to determine CFU per milliliter.

### Transposon library generation, screening of mutant library, and determination of insertion site.

The EZ-Tn5 <KAN-2>Tnp transposome kit (Epicentre Biotechnologies, Madison, WI, USA) was used to generate a transposon library in the E. coli parent strain according to the manufacturer's instructions. The E. coli parent strain was made competent for transformation by electroporation using a protocol provided by Epicentre Biotechnologies, which was based on a published protocol (23). Cells were cultured overnight in no-salt LB broth, subcultured and grown to an OD of 0.6 to 0.75 at 600 nm, and then subjected to multiple washes with ice-cold, sterile, 10% glycerol to reduce volume, ionic strength, and conductivity of the final cell suspension. The cells were transferred into cold (−80°C) 1.5-ml microcentrifuge tubes and transferred immediately to a −80°C freezer.

For library generation, cells were thawed on ice, and 1 μl each of Epicentre EZ-Tn5 <KAN-2>Tnp transposome and Type One inhibition restrictor was added to 50 μl. Electroporation and recovery were performed according to the protocol described by Dower et al. (24). Cells were incubated on ice for 15 min, pipetted into a chilled 0.1-cm-gap electroporation cuvette, and exposed to a single 1.8-kV, 25-μF, 200-mΩ pulse from a Harvard Apparatus BTX 630 electroporation system. Cells were immediately transferred to 950 μl SOC medium (Bioline USA, Taunton, MA, USA) at room temperature and incubated at 37°C with shaking for 1 h. Following transformation, cultures were plated on LB agar plates containing kanamycin and incubated at 37°C overnight. The resulting bacterial lawn was harvested by washing with LB broth containing kanamycin; glycerol was added to 20%, and the library was stored at −80°C.

Bacteria were initially screened from the pooled E. coli transposon library by treatment with 48 μg/ml recombinant human CXCL10. Given the low yield of isolates in the initial screen, subsequent screening was performed using 48 μg/ml and 36 μg/ml CXCL10 in parallel in order to maximize the number of mutants with increased resistance to CXCL10 that were captured by the screening process. The transposon library was treated with CXCL10 reconstituted in sterile water and stabilized with 0.3% HSA or the vehicle alone for 2 h at 37°C with shaking, after which time serial dilutions were plated on LB agar for determination of CFU per milliliter after overnight incubation at 37°C. Fifteen viable transposon mutants were identified from this primary screen, designated Tnx1 to Tnx15. These isolated transposon mutants then underwent secondary susceptibility screening with either a lower concentration of CXCL10 (6 μg/ml) or the vehicle alone. Since the parent strain bacteria are not completely killed after exposure to 6 μg/ml CXCL10, performing secondary screening with this lower concentration allowed us to determine the relative level of resistance of each transposon mutant to CXCL10 compared to that of the E. coli parent strain.

Transposon insertion sites in the two CXCL10-resistant transposon mutants (Tnx6 and Tnx15) were determined by PCR as described in our previous work (2, 25), modified for use with the EZ-Tn5 <KAN-2>Tnp transposome kit. Chromosomal DNA isolated from each mutant was digested with Hinp1I restriction enzyme (New England BioLabs, Ipswich, MA, USA) and ligated to the described Y-linker. After purification of the resulting ligation product, Epicentre primers Kan-2 FP-1 (ACCTACAACAAAGCTCTCATCAACC) and Kan-2 RP-1 (GCAATGTAACATCAGAGATTTTGAG) were used to perform the initial PCR to enrich for single-stranded DNA fragments flanking the transposon insertion. PCR was performed as described previously (2) with an increase of extension time (72°C) from 1 to 2 min for the single- and double-stranded amplifications. The double-stranded amplification products were separated on a 1.2% agarose gel; visible products were excised and purified using the QIAquick gel extraction kit (Qiagen, Germantown, MD, USA). PCR was performed on these products using the Y-linker custom primer JZ-99 (ACTACGCACCGGACGAGACGT) (Integrated DNA Technologies, Coralville, IA, USA) and either Kan-2 FP-1 or Kan-2 RP-1. Conditions for this final PCR were as described for the previous PCR amplification but with a decrease in extension time from 2 min to 1 min and an increase in the number of cycles to 35. The amplification products were separated on a 1.2% agarose gel; visible products were excised and purified. Twenty nanograms of DNA per 100 bp of PCR product was submitted to the University of Virginia Biomolecular Research Facility for sequencing with primer Kan-2 FP-1 or Kan-2 RP-1, as determined by gel visualization.

### Gene complementation.

The E. coli JW0110-2 strain, a Δ*aceE*::*kan* mutant from the Keio collection, was obtained via the Coli Genetic Stock Center, and removal of the FLP recognition target was accomplished as detailed above. The resulting markerless deletion mutant, the Δ*aceE* strain, exhibited increased resistance to CXCL10 compared to the parent strain. For *aceE* complementation, the native E. coli parent strain *aceE* gene, including its native promoter and ribosomal binding site, was amplified using Phusion high-fidelity polymerase (New England BioLabs, Ipswich, MA, USA) according to the manufacturer's instructions, with primer aceE-CF8 containing an SphI restriction site (GACTAGGCATGCCCAGAAGATGTTGTAAATCAAGC) and primer aceE-CR8 containing a SalI restriction site (CTAGTCGTCGACTTTACCTCTTACGCCAGACG). PCR cycle conditions were as follows: 98°C for 30 s, 98°C for 10 s, 68°C for 15 s, and 72°C for 45 s (32 cycles) and then 72°C for 7 min. The *aceE* amplification products were doubly digested with SphI-HF and SalI-HF (New England BioLabs, Ipswich, MA, USA), purified, and ligated into pBR322 that had also been digested with SphI-HF and SalI-HF. The prepared vector/*aceE* insert was transformed into Alpha-select E. coli (Bioline, Taunton, MA, USA) for propagation. This *aceE* complementation vector (pUVA411) and the empty-vector control (pBR322) were isolated from Alpha-select transformants grown in the presence of ampicillin using the QIAprep Spin miniprep kit (Qiagen, Germantown, MD, USA). After the pUVA411 cloning site was sequenced to verify appropriate *aceE* insertion, pUVA411 and pBR322 were individually transformed into electrocompetent E. coli Δ*aceE* and parent strain bacteria as described above. Diagnostic PCR and sequencing of the plasmids were used to validate E. coli transformants.

### Determination of bacterial growth rates.

The E. coli parent strain and Δ*aceE* mutant were grown overnight in LB broth as described above. A sufficient volume of overnight culture was inoculated into fresh LB broth to achieve an OD of ≈0.01 to 0.03 at 600 nm. Optical density was measured and recorded every 30 min for 8 h and then again at 24 h. Growth rate was determined based on a semilogarithmic plot of the OD_600_ growth curve versus time and was calculated using the equation μ = lnOD_2_ − lnOD_1_/(*t*_2_ − *t*_1_), where μ indicates the growth rate and *t* denotes time.

### Silver-enhanced immunogold labeling and visualization by TEM.

A modified preembedding protocol was used to perform CXCL10 immunogold labeling with silver enhancement based on our previously published protocol (12). E. coli (≈1 × 10^9^ cells) was incubated in 10 mM potassium phosphate buffer containing 1% Trypticase soy broth with or without 48 μg/ml CXCL10 in individual wells of a 24-well plate (1-ml final volume). At defined time points, vehicle-treated and CXCL10-treated samples were harvested and prepared for transmission electron microscopy (TEM), as previously described (12) with minor modifications. Briefly, single immunogold labeling with silver enhancement was performed on vehicle-treated and CXCL10-treated bacteria at 30 min using an adapted preembedding protocol (Aurion, Wageningen, Netherlands) described in detail elsewhere (26). Ultrasmall (≤1.0-nm) gold-conjugated F(ab′)_2_ fragments of goat anti-murine antibody (Ab), acetylated bovine serum albumin, cold-water fish skin gelatin, R-Gent SE-EM electron-microscopy-grade silver enhancement mixture, and Embed 812 resin were all purchased from the same commercial source (Electron Microscopy Sciences, Hatfield, PA, USA) and used according to the manufacturer's instructions. Bacterial samples were fixed with 4% paraformaldehyde for 30 min at 4°C followed by aldehyde inactivation using 0.1% glycine in phosphate buffer (PB). Samples were permeabilized with 0.1% saponin in phosphate-buffered saline (PBS) and then incubated in blocking solution (0.2% acetylated bovine serum albumin, 0.1% cold-water fish skin gelatin, 5% normal goat serum in PB). Incubations with primary murine anti-human CXCL10 monoclonal Ab (R&D Systems, Minneapolis, MN, USA) and secondary gold-conjugated F(ab′)_2_ fragments of goat anti-murine Ab were performed overnight at 4°C in PBS supplemented with 0.2% acetylated bovine serum albumin and 0.1% saponin. Before silver enhancement with the R-Gent SE-EM silver enhancement mixture, postfixation with 2.5% glutaraldehyde was performed. Samples were subsequently treated with 0.5% osmium tetroxide in PB for 10 min at room temperature before being dehydrated sequentially in 40%, 60%, 80%, and then 100% ethanol and embedded in Embed 812 epoxy resin. Ultrathin sections (80 nm) obtained with a diamond knife (Diatome, Bienne, Switzerland) were placed onto 200-mesh copper grids and contrast stained with lead citrate and uranyl acetate (Electron Microscopy Sciences, Hatfield, PA, USA). Sections were examined using a JEOL 1230 transmission electron microscope (JEOL, Peabody, MA, USA) operated at 80 kV; digital images were captured using an SIA-12 16-megapixel slow-scan charge-coupled device (Scientific Instruments and Applications, Duluth, GA, USA). All electron microscopy studies were performed at the University of Virginia Advanced Microscopy Facility.

### Statistical analysis.

**FIG 2:**
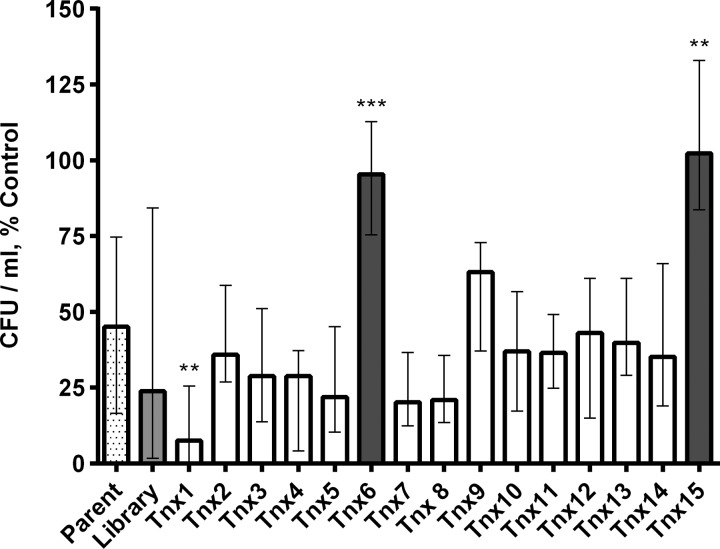
Secondary susceptibility screen of mutants isolated from E. coli transposon mutant library treated with CXCL10. A pooled E. coli transposon library of bacterial mutants was initially treated with 48 μg/ml; an additional two replications of this screen were repeated in parallel using either 48 μg/ml or 36 μg/ml recombinant human CXCL10 in 0.3% HSA or 0.3% HSA vehicle alone (untreated). Fifteen viable transposon mutants were isolated, designated Tnx1 to Tnx15. In a secondary screen of bacterial isolate resistance shown in this figure, transposon mutants Tnx1 to Tnx15 were individually examined for their level of CXCL10 resistance compared to that of the E. coli parent strain. Bacteria were treated with 6 μg/ml CXCL10 or 0.3% HSA vehicle alone for 2 h, and then serial dilutions were plated on LB agar for CFU-per-milliliter determination after overnight incubation at 37°C. Data are expressed as CFU per milliliter, percent control (a percentage of the viable CFU per milliliter for each specific isolate obtained by dividing the number of CFU per milliliter for the isolate after exposure to CXCL10 by the number of CFU per milliliter of that same isolate's corresponding untreated control). Bars represent median values ± ranges (*n* = 2 independent experiments performed in duplicate except for parent [*n* = 8], library [*n* = 3], Tnx6 [*n* = 5], and Tnx15 [*n* = 3]). Kruskal-Wallis test, *P* < 0.0001; Dunn posttest comparing each transposon mutant with CXCL10-treated parent strain bacteria (checkered bar), **, *P* = 0.001; ***, *P* = 0.0009. Isolates represented by open bars were not significantly resistant to CXCL10.

**FIG 3:**
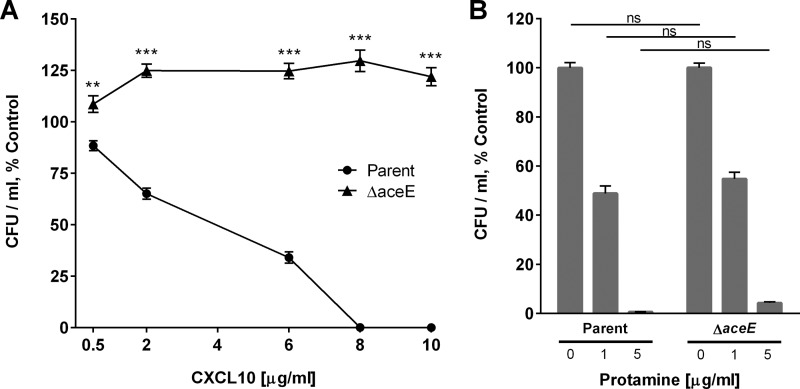
An E. coli *aceE* deletion mutant strain (Δ*aceE*) exhibits resistance to CXCL10-mediated antimicrobial activity. (A) The E. coli parent strain and the Δ*aceE* strain were treated with CXCL10 for 2 h, after which serial dilutions were plated on LB agar for CFU-per-milliliter determination following overnight incubation at 37°C. Reduction in bacterial viability is expressed as a percentage of untreated (vehicle-alone) control for each strain. Data points represent mean values ± standard errors of the means (*n* = 3 independent experiments performed in duplicate). The EC_50_ for CXCL10 against the E. coli parent strain (EC_50_, 2.8 μg/ml CXCL10; 95% CI, 2.5 to 3.2) was calculated as indicated in Materials and Methods. Statistical analysis revealed significant differences between the E. coli parent strain and the Δ*aceE* strain at all concentrations of CXCL10 tested. Two-way ANOVA, *P* < 0.0001; Bonferroni multiple-comparison test, **, *P* = 0.0002; ***, *P* < 0.0001. (B) The E. coli parent strain and the Δ*aceE* strain were treated with 1 μg/ml or 5 μg/ml protamine for 2 h, after which serial dilutions were plated on LB agar for CFU-per-milliliter determination following overnight incubation at 37°C. Reduction in bacterial viability is expressed as in panel A, with data points representing mean values ± standard errors of the means (*n* = 3 independent experiments performed in duplicate). Two-way ANOVA, *P* = 0.340; Bonferroni multiple-comparison test, ns, not significant.

**FIG 4:**
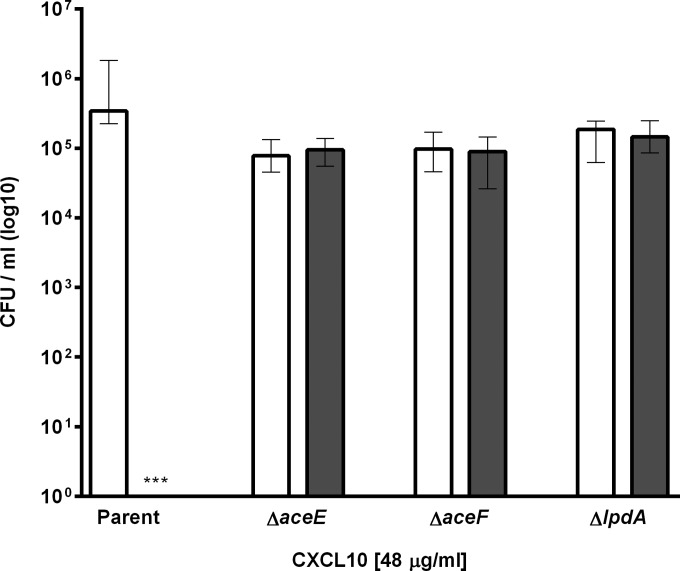
CXCL10 exhibits antimicrobial activity against the E. coli parent strain but not PDHc subunit deletion mutants. The E. coli parent strain and the PDHc subunit deletion mutants, the Δ*aceE*, Δ*aceF*, and Δ*lpdA* strains, were treated with 48 μg/ml CXCL10 or vehicle alone (untreated) for 2 h, after which samples were plated on LB agar to determine CFU per milliliter following overnight incubation at 37°C. Average initial inocula were 1.7 × 10^5^ for the parent strain, 1.9 × 10^5^ for the Δ*aceE* strain, 2.4 × 10^5^ for the Δ*aceF* strain, and 3.9 × 10^5^ for the Δ*lpdA* strain. Data are expressed as log_10_ CFU per milliliter, with open bars for untreated control and gray bars for CXCL10-treated groups. Bars represent median values ± ranges (*n* = 3 independent experiments with 3 replicates). Kruskal-Wallis test, *P* < 0.0001; Dunn posttest comparing untreated and CXCL10-treated groups of each strain, ***, *P* < 0.0001.

**FIG 5:**
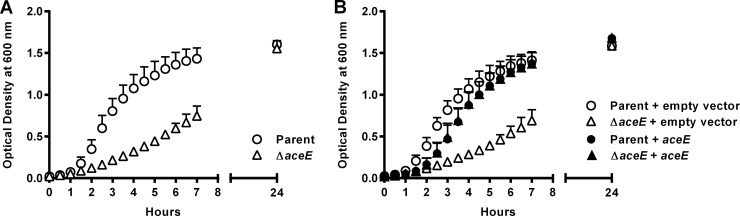
Complementation of E. coli Δ*aceE* with *aceE* restores an E. coli parent strain growth phenotype. (A) The OD at 600 nm was determined for cultures of the E. coli parent strain and the Δ*aceE* strain at set time points. The Δ*aceE* strain initially grew more slowly but reached optical densities similar to those of the parent strain at the later time point of 24 h. (B) The empty plasmid vector (pBR322) or the plasmid vector containing the native *aceE* gene along with its promoter and ribosomal binding site (pUVA411) was individually introduced into electrocompetent E. coli parent strain or Δ*aceE* bacteria; at set time points, OD was measured for cultures of each transformed strain. The growth curves for both the parent strain and the Δ*aceE* strain transformed with empty plasmid vector were unchanged from those of their respective originator strains in panel A (*P* = 0.9986 and *P* = 0.9142, respectively). In contrast, *aceE* complementation of the Δ*aceE* strain restored its growth rate to that of the *aceE*-complemented parent strain (*P* = 0.9852). A small but statistically significant difference was seen between the growth curves of the *aceE*-complemented parent strain or the *aceE*-complemented Δ*aceE* strain and the parent strain or the parent strain complemented with empty vector (*P* < 0.0001), likely due to overexpression of *aceE*. Despite this small difference, the growth curves of the Δ*aceE* strain and the Δ*aceE* strain complemented with empty vector remained significantly different from those of the parent strain and all other complemented strains (*P* < 0.0001). Data are expressed as OD at 600 nm; symbols represent each strain at a given time point ± standard deviation (*n* = 3 independent experiments per group).

**FIG 6:**
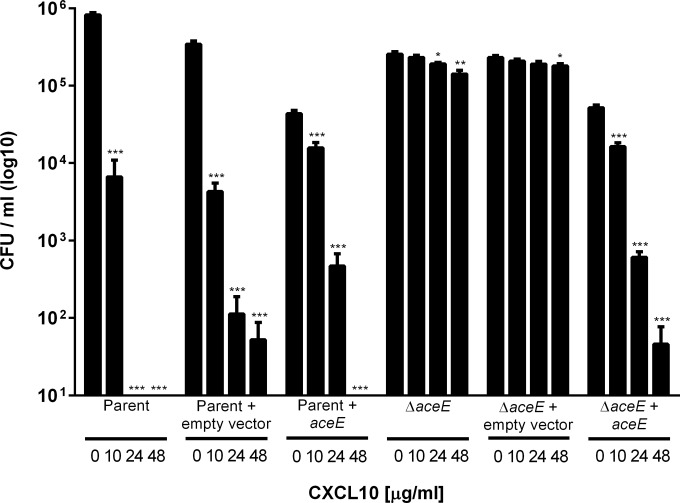
Complementation of E. coli Δ*aceE* with *aceE* restores the parent strain CXCL10 susceptibility phenotype. The E. coli parent strain remained similarly susceptible to increasing concentrations of CXCL10 when complemented with either empty vector (pBR322) or plasmid vector containing the *aceE* gene (pUVA411). E. coli Δ*aceE* remained resistant to the antimicrobial effects of CXCL10 when complemented with the empty vector but reverted to the parent strain susceptibility phenotype after complementation with plasmid vector containing *aceE*. E. coli parent strain bacteria, parent strain bacteria plus empty vector, parent strain bacteria plus *ace*-containing plasmid vector, Δ*aceE* strain bacteria, Δ*aceE* strain bacteria plus empty vector, and Δ*aceE* strain bacteria plus *ace*-containing plasmid vector were treated with increasing concentrations of CXCL10 for 2 h, after which serial dilutions were plated on LB agar for CFU-per-milliliter determination following overnight incubation at 37°C. Data are expressed as log_10_ CFU per milliliter; bars represent mean values ± standard errors of the means (*n* = 3 to 6 independent experiments per group, performed in duplicate). The one-way ANOVA *P* value was <0.0001 for each group tested except for the Δ*aceE* strain plus empty vector (*P* = 0.09). Dunnett's posttest analysis within groups showed susceptibility to increasing concentrations of CXCL10 compared with untreated controls as indicated above (***, *P* < 0.0001; **, *P* < 0.001; *, *P* < 0.05).

GraphPad Prism software version 4.0 or 6.0 (GraphPad Software, La Jolla, CA, USA) was used for statistical analysis and graphing. Significant differences in the levels of resistance to increasing concentrations of CXCL10 were detected between the E. coli BW25113 lab strain and CAV1036 (see Fig. 1) or between the BW25113 (parent) strain and Δ*aceE* mutant after exposure to CXCL10 or protamine (see Fig. 3) by two-way analysis of variance (ANOVA) with a Bonferroni multiple-comparison test. The fifty percent effective concentration (EC_50_) value for CXCL10 against the E. coli BW25113 lab strain and the CAV1036 clinical strain was determined by nonlinear regression analysis after transforming *X* values to log(*X*) and normalizing data to *Y* values of 0 to 100. Statistically significant differences in transposon mutants' resistance to CXCL10 relative to the parent strain during transposon library screening (see Fig. 2), as well as significant differences in CXCL10 resistance of pyruvate dehydrogenase complex (PDHc) deletion mutants compared to the parent strain (see Fig. 4), were determined using the Kruskal-Wallis test with Dunn posttest analysis. The Gompertz function with the least-squares method of fitting and the extra sum of squares F test were used to compare growth curves (see Fig. 5), while one-way ANOVA with Dunnett's posttest was performed for each group in the *aceE* complementation experiments (see Fig. 6). *P* values of <0.05 were considered statistically significant.

## RESULTS

### CXCL10 exerts an antimicrobial effect on both a K-12-derived laboratory strain of E. coli and a multidrug-resistant clinical strain, E. coli CAV1036.

To test the clinical relevance of the antimicrobial activity of CXCL10, we incubated both the E. coli parent strain and a multidrug-resistant E. coli clinical strain, CAV1036, with increasing concentrations of CXCL10. The CAV1036 strain, initially identified during an outbreak of infections with carbapenem-resistant Enterobacteriaceae at our hospital (19, 20), is a high-risk clone ST131 strain that carries the *bla*_KPC_ gene encoding Klebsiella pneumoniae carbapenemase and demonstrates resistance by broth microdilution not only to meropenem and imipenem but also to aztreonam, cefepime, piperacillin-tazobactam, ciprofloxacin, trimethoprim-sulfamethoxazole, and tetracycline (27).

Both the E. coli BW25113 lab strain and the CAV1036 clinical isolate were killed following incubation with concentrations of CXCL10 ranging from 10 μg/ml to 48 μg/ml (Fig. 1). The CXCL10 EC_50_ was 2.8 μg/ml (95% confidence interval [CI], 2.6 to 3.1) for the E. coli BW25113 lab strain and 8.6 μg/ml (95% CI, 7.9 to 9.3) for the CAV1036 clinical isolate. Incubation of the CAV1036 strain with lower concentrations of CXCL10 from 0.5 to 8 μg/ml for 2 h resulted in >100% viability, a percentage based on the comparison of CFU-per-milliliter results for CXCL10-treated CAV1036 to the CFU-per-milliliter results for the untreated CAV1036 strain. Microscopic examination of untreated CAV1036 revealed that this clinical isolate formed long chains of bacteria, but exposure to lower, sublethal concentrations of CXCL10 resulted in separation of the long chains into multiple, shorter chains of bacteria as well as individual bacteria that exhibited a tendency to form aggregates in the wells. Thus, the lower, sublethal concentrations of CXCL10 (0.5 to 8 μg/ml) had a notable effect on the morphology of the CAV1036 clinical isolate that impacted the percent viability measurements, whereas CXCL10 concentrations above 8 μg/ml exhibited lethal effects on the cells.

### Generation and screening of a transposon mutant library to detect E. coli isolates resistant to CXCL10-mediated antibacterial effects.

Utilizing an approach similar to that used by our laboratory to identify targets of chemokine-mediated antimicrobial activity in B. anthracis, we generated a transposon mutant library from the E. coli parent strain. The resultant pooled library of transposon mutants was screened for resistance to CXCL10 by incubation with 48 μg/ml (5.6 μM) of the chemokine. Due to the low number of isolates initially detected, further screening was performed twice more, in parallel with either 48 μg/ml or 36 μg/ml (4.2 μM) CXCL10, to maximize the number of resistant isolates detected. Of note, the E. coli parent strain is consistently killed at either of these concentrations of CXCL10. Fifteen transposon mutants, designated Tnx1 to Tnx15, were isolated from this primary screen.

Following the primary screen of the transposon mutant library, a secondary screen was performed in which the CXCL10-resistant transposon mutants Tnx1 to Tnx15 were isolated and individually tested for susceptibility to CXCL10. In the secondary screen, each isolate was incubated with a lower concentration of chemokine (6 μg/ml). Since the parent strain is not entirely killed by this CXCL10 concentration, secondary screening allowed us to compare the relative levels of susceptibility to CXCL10 between each of the selected transposon mutants and the parent strain (Fig. 2). Two transposon mutants, Tnx6 and Tnx15, exhibited significantly increased resistance to CXCL10 in relation to the parent strain. Therefore, these two mutants were chosen for further investigation.

### Identification of *aceE* gene disruption in CXCL10-resistant Tnx isolates.

We determined the transposon insertion site and identified the interrupted gene(s) using previously described methods adapted for use with the EZ-Tn5 <KAN-2>Tnp transposome (2, 25). After isolating and digesting chromosomal DNA from Tnx6 and Tnx15, we ligated the DNA digests with a partially double-stranded Y-linker. The ligation product was purified, after which initial PCR enrichment for single-stranded DNA segments flanking the transposon insertion was performed using Epicentre Kan-2 FP-1 and Kan-2 RP-1 primers. Two unique insertion sites were identified, both of which resulted in the disruption of a single gene, *aceE*, which encodes the E1 subunit of E. coli PDHc (28).

### Testing of susceptibility of Δ*aceE* and other PDHc subunit deletion mutants to CXCL10.

To further explore the role of *aceE* in the susceptibility of E. coli to CXCL10, we obtained an E. coli BW25113-derived *aceE* deletion mutant (JW0110-2) from the Keio Collection (21) via the Coli Genetic Stock Center. Following removal of the kanamycin resistance cassette as detailed above, the susceptibility of this Δ*aceE* strain was compared to that of the E. coli parent strain at increasing concentrations of CXCL10. The E. coli parent strain was killed after incubation with 8 μg/ml CXCL10 for 2 h (EC_50_, 2.8 μg/ml CXCL10; 95% CI, 2.5 to 3.2), while the Δ*aceE* strain, similar to Tnx6 and Tnx15, was significantly more resistant to killing by CXCL10 (Fig. 3A). To test if the observed resistance of the Δ*aceE* strain to CXCL10 was an effect specific to this chemokine rather than a more generalized effect that might apply to a cationic antimicrobial peptide, the parent strain bacteria and the Δ*aceE* strain were incubated with either a 1-μg/ml or a 5-μg/ml concentration of the cationic antimicrobial peptide protamine. The concentrations of protamine used were based on our prior testing of protamine against B. anthracis (2). Both the E. coli parent strain and the Δ*aceE* strain were similarly susceptible to killing by protamine (Fig. 3B).

To determine whether the killing effect of CXCL10 was dependent upon the presence of the *aceE* gene product E1p alone, or the functional complex PDHc, we obtained additional Keio collection deletion mutant strains lacking either *aceF* or *lpdA* (Keio strain designations JW0111-2 and JW0112-3, respectively), which encode the other subunits of PDHc (E2p and E3p, respectively). All three deletion mutant strains were found to be similarly resistant to the antimicrobial effects of CXCL10, even after exposure to chemokine concentrations as high as 48 μg/ml (Fig. 4). Taken together, these data suggest that the disruption of a functional PDHc, rather than that of *aceE* and the resultant enzyme subunit that it encodes, confers increased resistance to killing by CXCL10.

### Restoration of the parent strain E. coli CXCL10 susceptibility phenotype in the Δ*aceE* strain.

The correlation between disruption of *aceE* and the observed increase in CXCL10 resistance was tested through *aceE* complementation studies. Comparison of the growth curves of the E. coli parent strain and the Δ*aceE* strain revealed that the Δ*aceE* strain exhibited a lower growth rate (0.66 h^−1^ versus 1.28 h^−1^, respectively) (Fig. 5A). Complementation with an *aceE*-containing plasmid vector restored growth of the Δ*aceE* strain to nearly wild-type levels (Fig. 5B). In contrast, complementation of the Δ*aceE* strain with empty vector failed to significantly alter the growth rate from that of the uncomplemented Δ*aceE* strain (Fig. 5B). A small, but statistically significant, difference in growth of the strains complemented with *aceE* (both with growth rates of 0.96 h^−1^) was noted when they were compared to the parent strain alone (1.28 h^−1^) or parent strain complemented with empty vector (1.08 h^−1^), which was most likely due to overexpression of *aceE*. Complementation of E. coli Δ*aceE* with *aceE* decreased resistance to CXCL10 to a level equivalent to that of the parent strain, whereas there was no change in susceptibility phenotype of either the parent or *aceE* deletion mutant strain complemented with empty vector or the E. coli parent strain complemented with *aceE* (Fig. 6).

### Localization of CXCL10 to the bacterial cell surface of both E. coli parent strain and Δ*aceE* mutant.

**FIG 7:**
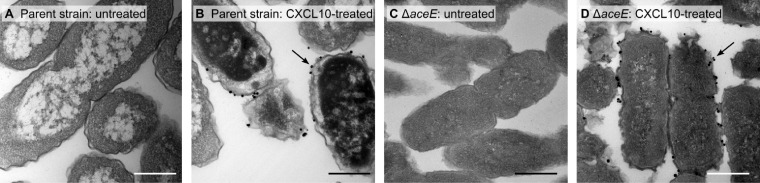
CXCL10 localizes to the bacterial surface in E. coli parent strain and Δ*aceE* strain bacteria. Bacteria were exposed to CXCL10 at a concentration of 48 μg/ml for 30 min. CXCL10 localization was then determined using silver-enhanced immunogold labeling of CXCL10 and visualized by TEM. (A) Untreated E. coli parent strain. (B) CXCL10-treated parent strain bacteria demonstrated localization of labeled gold particles to the bacterial membrane, as identified by black arrows. (C) Untreated E. coli Δ*aceE*. (D) CXCL10-treated Δ*aceE* bacteria demonstrated localization of CXCL10 to the bacterial membrane, as identified by black arrows, similarly to CXCL10-treated parent strain bacteria. Representative fields from 2 separate experiments, in which multiple images were obtained, are shown at ×30,000 magnification. Bars, 0.5 μm.

Silver-enhanced immunogold labeling of CXCL10 was performed to determine chemokine localization following incubation with either E. coli parent strain or Δ*aceE* bacteria. PDHc is thought to reside within the bacterial cytosol (28); however, immunogold-labeled CXCL10 was not observed within the cytosol of either strain of E. coli under the conditions used. Instead, in both the parent strain and the Δ*aceE* mutant, electron microscopy studies revealed localization of CXCL10 to the bacterial cell surface (Fig. 7). These data suggest that CXCL10 initially interacts with a bacterial surface component rather than directly with PDHc.

## DISCUSSION

This study establishes the importance of specific bacterial components in the *in vitro* CXCL10-mediated killing of E. coli. Through generation and screening of an E. coli transposon mutant library, we isolated two transposon mutants with significantly increased resistance to CXCL10-mediated killing. Both mutants contained unique disruptions within the gene *aceE*, which encodes E1p, the E1 subunit of PDHc.

PDHc is a large enzyme complex, which consists of multimers of three distinct subunits, E1p, E2p, and E3p (encoded by the genes *aceE*, *aceF*, and *lpdA*, respectively), all of which are required for its activity as a functional enzyme (29, 30). The ratio of subunit types, and hence the enzyme complex structure, differs between Gram-negative and Gram-positive bacteria, with the complex in the latter more closely resembling PDHc found in eukaryotic cells (29, 31). Through catalyzing the conversion of pyruvate to acetyl coenzyme A (acetyl-CoA), PDHc serves as an important link between glycolysis and the tricarboxylic acid (TCA) cycle, both of which function during aerobic respiration. Products of the TCA cycle, such as NADH, supply electrons for use within the electron transport chain, generating the proton motive force to drive ATP synthesis, flagellar rotation, and H^+^-dependent transporters (32, 33). In addition to its central role within the TCA cycle, acetyl-CoA is also required in the first committed step in fatty acid synthesis, the conversion of acetyl-CoA to malonyl-CoA by acetyl-CoA carboxylase (34).

Although disruptions in *aceF* and *lpdA* were not identified from screening of our transposon mutant library, both Δ*aceF* and Δ*lpdA* strains exhibited increased resistance to CXCL10-mediated antimicrobial activity at a level comparable to what was observed with *aceE* transposon and deletion mutants. These results implicate a functional PDHc rather than an individual PDHc subunit as the critical component necessary for CXCL10-mediated killing of E. coli. Importantly, the finding that the E. coli parent strain and the Δ*aceE* mutant were similarly susceptible to killing by the cationic antimicrobial protein protamine suggests that the primary mechanism of killing by CXCL10 is different from that of a cationic antimicrobial peptide. These data similarly discount the idea that the absence of the *aceE* gene product, E1p, or of a functional PDHc causes a change in membrane structure or function that results in a generalized resistance to bactericidal peptides.

Although PDHc is located in the bacterial cytosol, immunogold electron microscopy studies to evaluate localization of CXCL10 revealed that the chemokine appears to be localized to the bacterial surface of both the E. coli parent strain and the Δ*aceE* mutant. This localization suggests that the antibacterial effect of CXCL10 may be initiated by interaction of the chemokine with some common moiety on the bacterial cell surface and that killing is likely not due to a direct interaction with E1p. When taken together with our finding that all three PDHc subunit deletion mutants are similarly resistant to CXCL10-mediated killing, it supports the possibility that the antibacterial effect of the chemokine may be due to downstream effects on, or consequences of, an intracellular metabolic process(es). Alternatively, the lower growth rate of PDHc subunit deletion mutants might play an as-yet-undetermined role in conferring resistance to CXCL10-mediated killing. In the event that a cell surface target or targets exist, mutations in the genes encoding such components may be lethal, which would have prevented their detection during screening of the transposon mutant library.

Under aerobic conditions such as those used in our experiments, PDHc provides the primary means for production of acetyl-CoA, which, as noted above, serves as a central component for many different metabolic processes, including the TCA cycle; the glyoxylate shunt; glycolysis; gluconeogenesis; and lipopolysaccharide, peptidoglycan, and fatty acid synthesis (35). Two additional pathways for the production of acetyl-CoA have been described, but the first, which utilizes the enzyme pyruvate formate-lyase (PFL), functions mostly under anaerobic conditions (35). The second pathway uses the enzyme pyruvate oxidase (PoxB) to convert pyruvate to acetate, which is then irreversibly converted to acetyl-CoA *in vivo* by AMP-forming acetyl-CoA synthase (AMP-ACS) (36). This second pathway is typically used by E. coli to produce acetyl-CoA during stationary phase or under microaerobic conditions, although when PoxB is overexpressed or constitutively expressed, the pathway can function as a less efficient substitute for PDHc (35, 36). It is possible that E. coli Δ*aceE* bacteria are somehow able to avoid CXCL10-mediated killing by directing energy production away from aerobic respiration and the TCA cycle and instead forcing it to proceed through other, less efficient, metabolic pathways. Interestingly, although all three of the genes encoding PDHc subunits are essential in E. coli when glucose is the only available source of carbon, none are essential when bacteria are grown on Luria-Bertani (LB) medium (32). Another possibility is that CXCL10-mediated antimicrobial activity may be dependent upon the presence of a product of one of these pathways, for example, a lipid produced from acetyl-CoA.

Taken together with the results of our prior work in B. anthracis, our present findings support the concept that interferon-inducible ELR^−^ CXC chemokines exhibit CXCR3-independent antimicrobial effects via interaction with key bacterial components. The presence of functional PDHc appears to be a determining factor for whether CXCL10 exerts an antibacterial effect against E. coli, although PDHc itself does not appear to be a direct target of CXCL10. Interestingly, our prior studies have found *ftsX*, a gene encoding the transmembrane domain of an ATP-binding cassette transporter, to be the primary component implicated in CXCL10-mediated killing of B. anthracis (2), indicating that this component or different bacterial components may be targeted by CXCL10 in susceptible Gram-negative and Gram-positive microorganisms. The existence of distinct bacterial targets of CXCL10 in Gram-positive and Gram-negative bacteria is further supported by differences in localization of immunogold-labeled CXCL10 to the bacterial cell surface. In the B. anthracis Sterne strain, CXCL10 localizes to the bacterial membrane but it does not localize to any structure of the *ftsX* deletion mutant strain. In contrast, in E. coli, CXCL10 localizes to the cell surface of both parent and Δ*aceE* strains, suggesting that the chemokine is interacting with an as-yet-unidentified moiety rather than directly with PDHc or one of its three enzyme subunits.

Further investigation into the mechanism(s) by which CXCL10 exerts its antimicrobial effect against E. coli not only should broaden our current understanding of the pleiotropic role of chemokines in host defense but may also reveal new drug targets that can be utilized to treat infections caused by E. coli or other antibiotic-resistant Gram-negative pathogens. Considering the critical lack of effective treatment options for infections caused by these bacteria, pursuing such avenues of inquiry will facilitate the development of novel therapeutic agents at a time when they are urgently needed.
